# Zhi‐zi‐chi decoction mitigates depression by enhancing lncRNA Six3os1 expression and promoting histone H3K4 methylation at the BDNF promoter

**DOI:** 10.1111/jcmm.18365

**Published:** 2024-05-31

**Authors:** Tianyu Zou, Kazuo Sugimoto, Yu Zhao, Baitao Li, Xiaomao Zhou, Cheng Peng

**Affiliations:** ^1^ Department of Encephalopathy Shenzhen Luohu District Hospital of Traditional Chinese Medicine Shenzhen China; ^2^ Department of Encephalopathy Shenzhen Hospital of Shanghai University of Traditional Chinese Medicine Shenzhen China; ^3^ Department of Neurology, Dongzhimen Hospital Beijing University of Chinese Medicine Beijing China; ^4^ Institute for Brain Disorders Beijing University of Chinese Medicine Beijing China; ^5^ Department of Acupuncture, First Affiliated Hospital Heilongjiang University of Chinese Medicine Harbin China

**Keywords:** anti‐depressive mechanism, BDNF, histone methylation, KMT2A, LncRNA Six3os1, neuron injury, traditional Chinese medicine, Zhi‐zi‐chi decoction

## Abstract

Traditional Chinese medicine, particularly Zhi‐zi‐chi decoction (ZZCD), is gaining recognition as a potential treatment for depression. This study aimed to uncover the molecular mechanisms behind ZZCD's antidepressant effects, focusing on lncRNA Six3os1 and histone H3K4 methylation at the BDNF promoter. Network pharmacology and in vivo experiments were conducted to identify ZZCD targets and evaluate its impact on depression‐related behaviours and neuron injury. The role of Six3os1 in recruiting KMT2A to the BDNF promoter and its effects on oxidative stress and neuron injury were investigated. ZZCD reduced depression‐like behaviours and neuron injury in mice subjected to chronic stress. It upregulated Six3os1, which facilitated KMT2A recruitment to the BDNF promoter, leading to increased histone H3K4 methylation and enhanced BDNF expression. ZZCD also inhibited CORT‐induced neuron injury, inflammatory response and oxidative stress in vitro. ZZCD's antidepressant properties involve Six3os1 upregulation, which exerts neuroprotective effects by inhibiting oxidative stress and neuron injury, thereby alleviating depressive symptoms. Targeting Six3os1 upregulation may offer a potential therapeutic intervention for depression.

## 1 INTRODUCTION

Depression is considered to be a prevalent psychiatric illness, with a high prevalence worldwide.^1^, ^2^ A range of neurobiological, genetic and environmental factors are involved in the onset, maintenance and progression of depression.^3^ Investigating these mechanisms can provide a greater understanding of the underlying biological mechanisms and identify potential biomarkers or therapeutic targets for the development of more effective interventions.^4^ Moreover, examining the complex interplay between genetic and environmental factors in the development of depression can provide insights into potential risk factors, such as early life stress, that may contribute to the development of depression.^5^ A greater understanding of these mechanisms can lead to the development of more personalized interventions that target specific biological pathways and provide a more tailored approach to depression treatment.^6^ Traditional Chinese medicine has been used as a therapy to treat neuropsychiatric diseases over a long period in China.^7^ TCM characterizes depression as a result of imbalances in the body's Qi and organ systems, and practitioners aim to identify the root cause of these imbalances through various diagnostic techniques.^8^ TCM approaches treatment through various modalities, such as herbal medicine, acupuncture, dietary therapy and mind–body practices.^9^ Investigating TCM for depression can highlight the benefits of a holistic and integrative approach to treating mental health conditions.^10^ Moreover, traditional Chinese medicine can offer non‐pharmacological and non‐invasive treatment options for those inclined towards non‐pharmacological therapies, such as acupuncture, dietary therapy and Tai Chi.^11^

Recent studies have shown that individuals with depression exhibit higher levels of neuroinflammation compared to the control group. This neuroinflammation is predominantly observed in the prefrontal cortex, anterior cingulate cortex and hippocampus.^12^ Consequently, the neuroinflammation and associated pro‐inflammatory cytokines TNF‐α, IL‐1β and IL‐6 contribute to the impairment of hippocampal neurogenesis, potentially facilitating depressive‐like behaviour.^13^, ^14^ Zhi‐zi‐chi Decoction (ZZCD), consisted of *Fructus Gardeniae* (Zhizi in Chinese, ZZ) and *Semen sojae praeparatum* (Dandouchi in Chinese, DDC), is a traditional Chinese medicine formula and exerts anti‐depressive effects by suppressing neuron injury in glutamate‐treated PC12 cells and depression‐like behaviours in chronic unpredictable mild stress (CUMS)‐induced rats.^15^ In addition, another study also indicated that ZZCD exerts potential antidepressant effects by reversing the imbalance of glutathione and oxidative stress in the brain of CUMS‐induced rats with depression‐like behaviours.^16^ Geniposide, an iridoid glycoside derived from *Fructus Gardeniae*, has been reported to upregulate the expression of long non‐coding RNA (lncRNA) Six3os1 in CUMS‐induced neurons.^17^ In NH4Cl‐treated hippocampal neurons, the dendritic spine density and maturity are decreased, while silencing of Six3os1 can reverse this decrease.^18^ Additionally, Six3os1 upregulation is capable of ameliorating the oxidative stress of neurons in CUMS‐induced mice with depression‐like behaviours.^17^

Six3os1 can bind to lysine (K) methyltransferase 2a (KMT2A), as predicted by the catRAPID website. KMT2A is known as a regulator of histone H3 lysine 4 (H3K4) methylation, a histone modification enriched at promoters and enhancers, abundantly expressed throughout the brain.^19^ The enhanced binding of KMT2A to the brain‐derived neurotrophic factor (BDNF) promoter region 2 due to chronic morphine exposure is associated with increased H3K4me3 levels.^20^ BDNF is a neurotrophin widely expressed in the brain regions, including cortex, cerebellum, hippocampus and basal forebrain.^21^ As reported, the regulation of BDNF is essential for the pathophysiology of depression and related brain functions in which epigenetic alterations of the BDNF gene may be involved; BDNF methylation has the potential to be a biomarker of depression.^22^, ^23^

Herein, in light of the aforementioned findings, we might hypothesize that ZZCD could alleviate depression, acting in association with regulation of the Six3os1/KMT2A/BDNF axis. Therefore, we undertook a series of experiments in the CUMS mouse model and corticosterone (CORT)‐stimulated hippocampal neurons, with the aim to illuminate the molecular mechanism for ZZCD in the treatment of depression. These insights into the mechanisms underlying the effectiveness of ZZCD can lead to the development of more targeted and effective therapies for depression.

## 2 MATERIALS AND METHODS

### 2.1 ETHICS STATEMENT

The current study was performed with the approval of the Animal Ethics Committee of Shenzhen Luohu District Hospital of Traditional Chinese Medicine (20210901003) and performed in strict accordance with the Guide for the Care and Use of Laboratory animals published by the US National Institutes of Health.

### 2.2 Network pharmacology and in silico analysis of targets

The main active ingredients of ZZ and DDC in ZZCD were retrieved from the TCMSP database and screened with oral bioavailability (OB) >30% and drug‐like (DL) properties >0.1 as the threshold. The targets of these active ingredients were downloaded from TCMSP database. Next, depression‐related targets were searched from the GeneCards and DisGeNET databases, followed by intersection analysis using R ‘venn’ package. Next, the disease targets were intersected with the targets of active ingredients.

Network diagram of the ‘active ingredient‐target’ was plotted by Cytoscape software. An interaction network involving was then drawn using STRING database, with ‘mus musculus’ as the species. The obtained network was imported into Cytoscape software, and each node gene was scored using the CytoNCA plug‐in. Genes were filtered according to the median value of Betweenness, Closeness, Degree, Eigenvector, LAC and Network to obtain the candidate target genes. The candidate target genes were then subjected to ID conversion using the R ‘org.Hs.eg.db’ package, followed by KEGG pathway enrichment analysis using the R ‘ggplot2’ and ‘ClusterProfiler’ packages.

Depression‐related dataset GSE84183 was retrieved from GEO database, which contains 64 samples. Of them, eight normal hippocampal tissue samples and eight CUMS hippocampal tissue samples were selected for differential analysis using the R ‘limma’ package, with adj.*P*. A value <0.05 as the threshold. A box plot of the differentially expressed genes was then plotted using the R ‘ggplot2’ software package.

The binding between lncRNA Six3os1 and BDNF, KMT2A was predicted by the catRAPID website. The localization of lncRNA Six3os1 in the cells was predicted by the lncLocator website.

### 2.3 Histone modification sites and histone methyltransferase acquisition

Histone H3K4 methylation‐binding site in the BDNF promoter region was searched through the ENCODE and UCSC websites. H3K4 histone methyltransferase, genes regulating histone H3K4me3 and genes associated with H3K4 methylation activation were searched by the GSEA‐MSigDB database, followed by intersection analysis using the jvenn website to obtain candidate genes encoding H3K4me3 transferase.

### 2.4 Preparation of ZZCD

Dried ripe fruits of *Fructus Gardeniae* (180525) and fermented ripe seeds of DDC (180716‐1) were purchased from Shanghai Tong Han Chun Tang Chinese Herbal Medicine Factory (Shanghai, China). In total, 50 g of ZZ and 50 g of DDC were extracted twice by refluxing with 800 mL of 50% ethanol for 1 h. Then, the filtrate was mixed and centrifuged at 3000 ***g*** for 10 min. After the supernatant was collected, the crude extract was prepared by evaporating to dryness by rotary vaporization at 60°C. The crude extract was dissolved and loaded on D101 macroporous adsorption resin for 2 h. The resin was rinsed in different concentration of ethanol (0, 10%, 20%, 30%, 40%). Only the 40% ethanol eluent was collected, concentrated by evaporation and dried to extract powder. For animal experiments, the extract powder was prepared into high‐, medium‐ and low‐dose concentrations (10, 5 and 2.5 g/kg, respectively, medium dose means the routine dose). For cell experiments, the extract powder was prepared to a concentration of 100 mg/mL and stored at −80°C.^15^

### 2.5 Identification of ZZCD extract using UPLC–MS

Quantitative analysis and pharmacokinetic studies of the ZZCD extract were conducted on the Shimadzu LC–MS (8040) system, equipped with two LC‐30A pumps, an SIL‐30AC autosampler, a CTO‐30A column oven and an LCMS‐8040 triple quadrupole mass spectrometer, and the Shimadzu LC–MS/MS (8045) system, equipped with the same components as the LC‐MS (8040). The analysis was performed using a Shimadzu Shim‐pack GIST C18 column (2.1 × 100 mm, 2 μm), with a flow rate of 0.4 mL/min at 40°C. The mobile phase consisted of 0.1% acetic acid solution (A) and acetonitrile (B), following a linear gradient programme as follows: 0–2 min, 5% B; 2–5 min, 5%–15% B; 5–10 min, 15%–80% B; 10–12 min, 80% B; 12–13 min, 80%–5% B; 13–15 min, 5% B. A sample injection volume of 2 μL was used for quantitative analysis of the ZZCD extract and 5 μL for pharmacokinetic studies. The mass spectrometer was equipped with an electrospray ionization source (ESI) and multiple reaction monitoring (MRM) mode to obtain chromatograms for positive and negative ionization. The final conditions were set as follows: spray gas flow rate at 3 L/h, drying gas flow rate at 15 L/h, desolvation line (DL) temperature at 250°C, heat block temperature at 400°C and collision gas (argon) at 230 kPa. For the quantitative analysis of compounds, the following MRM transitions were used: rhaponticin at 433.15/271.15, liquiritin at 477.20/285.10, daidzin at 417.20/255.10, geniposide at 447.30/225.25, genipin gentiobioside at 609.30/549.25, 6‐α‐hydroxygenipin at 463.30/241.40, geniposide methyl ester and gardenoside at 463.30/241.45 and paeoniflorin (IS) at 539.30/449.20. To identify the main metabolites of the ZZCD extract, including genipin and genipin sulfate conjugates, XSelect HSS T3 column (2.1 mm × 100 mm, 2.5 μm) was employed on the Shimadzu HPLC‐MS (8045) system, with a flow rate of 0.3 mL/min and temperature set at 40°C. The elution was completed using a linear gradient programme with a combination of 0.1% formic acid‐water (A) and 0.1% formic acid‐acetonitrile (B) as the mobile phase (0–2 min, 5% B; 2–13 min, 5%–95% B; 13–15 min, 95% B). The injection volume was 3 μL. Genipin and genipin sulfate conjugates were detected in negative electrospray ionization in the full scan mode at m/z 200–600. The MS parameters were as follows: capillary voltage at 3500 V, nebulizing gas pressure at 35 psi, drying gas at 11 L/min, drying gas temperature at 350°C and fragment voltage at 120 V. The UPLC‐MS chromatograms and concentration determination of the effective fraction of ZZCD extract are shown in Figure S1 and Table S1.^24^

### 2.6 Construction of CUMS‐induced mouse models of depression

A total of 120 healthy C57BL/6N male mice (aged 6–8 weeks; Beijing Vital River Laboratory Animal Technology Co., Ltd., Beijing, China) were housed individually in the SPF laboratory at 25–27°C and 45%–50% humidity under a 12‐h light/dark cycle for 1 week.

These mice were divided into control (*n* = 12) and CUMS groups (*n* = 108). Control mice were injected with sh‐NC + oe‐NC plasmids. The CUMS mice were further treated with ZZCDL (2.5 g/kg low dose), ZZCDM (5 g/kg medium dose), ZZCDH (10 g/kg, high dose), sh‐NC + oe‐NC (injected with sh‐NC + oe‐NC plasmids), ZZCD + sh‐NC + oe‐NC (treated with 5 g/kg ZZCD and injected with sh‐NC + oe‐NC plasmids), ZZCD + sh‐Six3os1 + oe‐NC (treated with 5 g/kg ZZCD and injected with sh‐Six3os1 + oe‐NC plasmids) and ZZCD + sh‐Six3os1 + oe‐BDNF (treated with 5 g/kg ZZCD and injected with sh‐Six3os1 + oe‐BDNF plasmids), *n* = 12 for each treatment. sh‐Six3os1 and sh‐NC plasmids were designed by the BLOCK‐iT™ RNAi Designer software (Invitrogen, Thermo Fisher Scientific) and constructed by Thermo Fisher Scientific.

With the exception of those in the control and sh‐NC + oe‐NC group, all mice were subjected to CUMS protocol for 8 weeks (Table S2).^15^ A stress method was randomly selected every day, so that the mice could not predict the stress form. The same stress could not appear continuously, and all mice received the same operation on the test day. After 6 weeks of CUMS modelling, mice following each treatment were intragastrically administered ZZCD once a day, and 3 days before the end of administration, 10 μg of sh‐Six3os1, oe‐BDNF, oe‐NC or sh‐NC plasmids were injected into the right ventricle of mice at a rate of 0.5 μL/min within 10 min using a stereotaxic instrument,^25^ once a day for 3 consecutive days. At 8 weeks of CUMS, mice were weighed and behavioural tests were conducted to verify the success of the modelling. Mice were deeply anaesthetised by inhalation of 3% isoflurane (792632, Sigma), after which blood samples were collected by orbital puncture and centrifuged to harvest serum. Hippocampal tissues were collected, frozen in liquid nitrogen and stored at −80°C.

### 2.7 Behavioural tests

In sucrose preference test (SPT), mice were subjected to food and water deprivation for 24 h and then provided with a bottle of 1% sucrose water and a bottle of pure water. After 24 h, the percentage of sucrose preference was calculated by measuring the consumption of sucrose water and pure water as follows: sucrose preference percentage (%) = (sucrose water consumption/total liquid consumption) × 100%.

In tail suspension test (TST), mice were suspended 25 cm off the ground, with their tail tips (1 cm) tied to a horizontal line. Immobility time was recorded during the 6‐min test period (the first 1 min was used for acclimatization, and the remaining 5 min was recorded). A mouse was considered immobile only when it was suspended passively and fully suspended. The immobility time (%) of mice in the following 5 min was measured.

In forced swimming test (FST), a media cylinder with base (20 cm in diameter and 50 cm in height) was used. The water temperature was 23–25°C and the water depth was kept that the tip of mouse tail failed to touch the bottom of the container. On Day 1, mice were given 15 min pre‐swimming training and a 5‐min forced swimming test was performed on Day 2, with the immobility time recorded.

In open field test (OFT), a customized plastic box (40 cm × 40 cm × 30 cm) was equipped with a video‐tracking system (Smart 3.0, Panlab). Mice were placed in the central square for 10 min, and the time spent in the central area (20 cm × 20 cm) was automatically recorded.

### 2.8 ELISA

Mouse hippocampal tissue samples (0.5 g) were lysed with 300 μL of tissue lysis solution to extract protein and incubated on ice for 30 min. The homogenate was centrifuged at 13,000 ***g*** and 4°C for 15 min, with the supernatant transferred to Eppendorf tubes. The total protein content in the tissue lysate was subsequently measured with a BCA kit (P0012S, Beyotime, Shanghai, China). Mouse serum samples were also collected. Next, SOD, MDA, GSH and CAT were measured in the tissue supernatant and serum using their separate kits (SOD, S0109, Beyotime; MDA, S0131S; GSH, S0057S; CAT, S0051). Levels of inflammatory factors TNF‐α, IL‐1β and IL‐6 in the tissue supernatant and serum were determined using their separate kits (TNF‐α, PT512, Beyotime; IL‐1β, PI301; IL‐6, PI326). The oxidative stress indicators and inflammatory factor levels in the cell supernatant were measured using the same way as in the mouse serum.^26^

### 2.9 HE staining

Coronal hippocampal sections (4 μm) were heated at 60°C for 1 h, dewaxed in xylene and dehydrated in ascending series of alcohol. Next, the sections were stained with haematoxylin (G1140, Solarbio, Beijing, China) for 2 min, hydrolysed by 1% hydrochloric acid‐ethanol for 10 s, counterstained with eosin (G1100, Solarbio) for 1 min, dehydrated, cleared and mounted before observation under an optical microscope (XP‐330, Shanghai Bingyu Optical Instrument Co., Ltd., Shanghai, China). The stained sections were scored by two experienced pathologists in a blinded fashion using a 4‐point scale (0, normal; 1, mild; 2, moderate; and 3, severe).^27^

### 2.10 Nissl staining

Paraffin sections were immersed in 0.1% cresyl violet (C5042, Sigma) at 37°C for 15 min for Nissl staining. Only neurons with Nissl and intact morphology were regarded as surviving cells. Cell counts were performed on five randomly selected non‐overlapping areas in the hippocampal CA1 region of each slide. The survival index was defined as follows: survival index (%) = 100 × (number of surviving neurons/total number of neurons). The stained cells were observed and captured using an optical microscope.^28^

### 2.11 TUNEL staining

In situ cell death detection kit (KHO1001, Thermo Fisher Scientific) was used for cell apoptosis determination. Paraffin sections of mouse hippocampal tissues were permeabilized with Triton X‐100, treated with 3% H_2_O_2_ and incubated with TUNEL reaction mixture and transforming peroxidase to label the broken DNA strands in apoptotic cells. Afterwards, sections were developed with DAB (DA1010, Solarbio), stained with haematoxylin, dehydrated, cleared and mounted before observation under an ordinary optical microscope. The number of TUNEL‐positive cells per unit area (nuclei of positive cells stained in dark brown) was calculated as the number of apoptotic cells.^29^

### 2.12 Primary hippocampal neuron culture for mimicking neuron injury in vitro

Primary hippocampal neurons were prepared from embryos of pregnant C57BL/6N mice on the 16–17 days. In brief, cerebral cortex was opened to expose hippocampal tissues, which were dissociated with 0.125% trypsin (25200114, Thermo Fisher Scientific) at 37°C for 15 min and this reaction was halted by addition of 4 mL of DMEM/F‐12 medium (11330032, Thermo Fisher Scientific) containing 20% FBS (16140063, Thermo Fisher Scientific). The resulting cell suspension was centrifuged at 150 ***g*** for 5 min and resuspended in DMEM/F‐12 containing 20% FBS. After passage through a 100‐mm sterile filter, cells were seeded on a sterile culture dish coated with poly‐L‐lysine (0.1 mg/mL, P7886, Sigma) and cultured at 37°C with 5% CO_2_. Three hours later, the medium was replaced by Neurobasal medium (A3653401, Thermo Fisher Scientific) supplemented with 2% B27 (A3653401, Thermo Fisher Scientific). AraC (10 mol/L, C6645, Sigma) was added to the medium to inhibit glial cell growth.

Primary hippocampal neurons were stimulated with 50 μM CORT (802905, Sigma) for 24 h to establish an in vitro neuron injury model.^30^

### 2.13 Immunofluorescence staining

Isolated primary hippocampal neurons were identified using immunofluorescence staining. Primary hippocampal neurons were fixed with 4% paraformaldehyde for 5 min, permeabilized with 0.5% Triton X‐100 in PBS for 15 min and blocked with 1% BSA at room temperature for 30 min. The cells were probed with primary antibody to neuronal dendritic marker protein MAP‐2 (1:200, #8707, Cell Signaling Technologies [CST], Beverly, MA) at 4°C overnight. The next day, cells were re‐probed with fluorescently labelled secondary antibody (1:200, #4412, CST) l h at room temperature, sealed and observed under a fluorescence microscope.^31^

### 2.14 Cell transfection

Six3os1 and BDNF overexpression plasmids (oe‐Six3os1 and oe‐BDNF) and their control oe‐NC were purchased from Guangzhou RiboBio Co., Ltd. (Guangzhou, Guangdong China) and constructed with pEXP‐RB‐Mam (R11091.1, RiboBio). shRNAs targeting Six3os1 (sh‐Six3os1) and BDNF (sh‐BDNF) along with their NC sh‐NC were designed by the BLOCK‐iT™ RNAi Designer software (Invitrogen, Thermo Fisher Scientific) and constructed by Thermo Fisher Scientific. The relevant sequences are shown in Table 3.

Primary hippocampal neurons at Passage 3 were transfected with oe‐NC, oe‐Six3os1, sh‐NC, sh‐Six3os1#1 and sh‐Six3os1#2. CORT‐stimulated neurons were transfected with oe‐NC + sh‐NC, oe‐Six3os1 + sh‐NC and oe‐Six3os1 + sh‐BDNF. In addition, CORT‐stimulated neurons were treated with sh‐NC + oe‐NC, ZZCD + sh‐NC + oe‐NC, ZZCD + sh‐Six3os1 + oe‐NC and ZZCD + sh‐Six3os1 + oe‐BDNF. After plasmid transfection for 24 h, these cells were stimulated with CORT for another 24 h and then treated with ZZCD (4 mg/mL) for 24 h.^15^ Transfection was conducted using Lipofectamine 2000 reagent (11668‐019, Invitrogen, Thermo Fisher Scientific).

### 2.15 CCK‐8 assay

Primary hippocampal neurons were seeded in 96‐well plates at a density of 8 × 10^3^ cells/well and incubated in an incubator for 24 h. Cells were treated with ZZCD at varied concentrations (0, 1, 2, 4, 6, 8 and 10 mg/mL) for 24, 48 and 72 h or with 50 μM CORT for 24 h and then with ZZCD at varied concentrations, followed by incubation with 10 μL CCK‐8 solution (96992, Sigma) for 1 h in a 37°C humidified incubator. The OD value of each well was measured at 450 nm using an Epoch microplate spectrophotometer (Bio‐Tek, Winooski, VT).

### 2.16 Flow cytometry

Cell apoptosis detection was performed using the flow cytometry kit (APOAF, Sigma). Hippocampal neurons were prepared into suspension (1 × 10^5^ cells/mL) and incubated with 5 μL FITC‐Annexin V and PI for 20 min at room temperature. Apoptosis of cells was then measured using a flow cytometer (Guava® easyCyte™ 6‐2L Base System, 0500‐5007, Luminex), with data analysed using CellQuest Pro software.

### 2.17 RT‐qPCR

Total RNA was extracted from neurons using TRIzol reagent (15596026, Invitrogen, Thermo Fisher Scientific), the concentration and purity of which were determined using NanoDrop One/OneC spectrophotometer (1011U, NanoDrop Technologies Inc., Wilmington). Total RNA was reversely transcribed into cDNA using PrimeScript RT reagent Kit (RR047A, Takara, Japan). RT‐qPCR was conducted using Fast SYBR Green PCR Kit (RR820A, Takara) and ABI PRISM 7300 RT‐PCR system. GAPDH was regarded as internal reference, and the fold changes were calculated by 2^−△△Ct^. The primer sequence is shown in Table 4.

### 2.18 Western blot

Total protein was extracted, separated and transferred onto membranes. The membrane was blocked using 5% skimmed milk powder and underwent overnight incubation at 4°C with primary rabbit antibodies to BDNF (1:1000, A1307, Abclonal, Wuhan, China), Histone H3 (1:1000, A2348, Abclonal), H3K4me3 (A2357, 1:1000, ABclonal) and β‐actin (1:1000, ab8226, Abcam). The next day, membrane was incubated with HRP‐labelled secondary antibody goat anti‐rabbit IgG (1:10000, BA1054, Boster Biological Technology Co. Ltd., Wuhan, Hubei, China) for 1 h at room temperature. Then, ECL reagent (AR1172, Boster) was used to visualize the immunocomplexes on the membrane, and band intensities were quantified using ImageJ software.

### 2.19 FISH assay

Ribo™ lncRNA Six3os1 FISH probe mixture (red) (RiboBio) was used for this assay. Primary hippocampal neurons were seeded into 6‐well culture plates covered with coverslips for 1 day to reach approximately 80% confluence. Next, cells were fixed in 1 mL of 4% paraformaldehyde at room temperature, treated with proteinase K (2 μg/mL), glycine and ethanolamine, followed by incubation with 250 μL prehybridization solution for 1 h at 4°C. Subsequently, 250 μL hybridization solution containing 300 ng/mL probe was added to the cells and hybridized overnight at 42°C. Afterwards, cells were stained with DAPI (1:800) diluted in PBST, seeded in 24‐well culture plates for 5 min and sealed with fluorescence decay resistant medium, and observed under a fluorescence microscope in five different fields of view.

### 2.20 RIP assay

The binding of Six3os1 to the KMT2A protein was detected using RIP kit (17‐701, Millipore, Billerica, MA). Primary hippocampal neurons were lysed on ice for 5 min with an equal volume of RIPA lysis buffer (P0013B, Beyotime) and centrifuged at 32841.32 g and 4°C for 10 min. The supernatant was harvested, a portion of which was removed as input and the other was probed with antibodies to Kmt2a (1:100, NB600‐248, Novus Biologicals) and IgG (1:100, ab172730, Abcam, taken as NC) for co‐precipitation. Finally, immunoprecipitated RNA were isolated and analysed by qPCR detection.

### 2.21 ChIP assay

Primary hippocampal neurons were treated with 1% formaldehyde and incubated for 10 min to generate DNA‐protein crosslinks. Then, the cell lysates were sonicated to generate chromatin fragments of 200–1000 bp and immunoprecipitated with KMT2A (1:100, NB600‐248, Novus Biologicals), H3K4me3 (1:100, A2357, ABclonal) and IgG (1:50, #3900, CST, taken as NC). The precipitated chromatin DNA was analysed by qPCR assay using iQ SYBR Green Supermix (Bio‐Rad, Hercules, CA). The primer sequence for the BDNF promoter was forward: 5′‐GTAAATGCTTACAAAATCGA‐3′ and reverse 5′‐CAAAGCTAGCCTATCCTACA‐3′.

### 2.22 RNA pull‐down

For the RNA pull‐down assays, biotinylated probes Bio‐probe‐NC, Bio‐Six3os1‐Wt and Bio‐Six3os1‐Mut (each at a concentration of 50 nM) were transfected into primary hippocampal neurons. After 48 h, cells were harvested and lysed by centrifugation. Subsequently, the cell lysates were incubated with cell lysis buffer for 10 min and then aliquoted into 50 mL samples. The remaining cell lysates were incubated with pre‐coated streptavidin magnetic beads (88816, Thermo Fisher Scientific, USA) at 4°C for 3 h, followed by two washes with cold lysis buffer, three washes with low‐salt buffer and one wash with high‐salt buffer. Finally, RNA bound to the beads was purified using Trizol, and the enrichment of the Kmt2a promoter was assessed by RT‐qPCR.^17^

### 2.23 Statistical analysis

Statistical comparison was performed using unpaired *t*‐test when only two groups were compared, or by Tukey's test‐corrected one‐way ANOVA or repeated measures ANOVA when more than two groups were compared. All statistical analyses were completed with SPSS 21.0 software (IBM, Armonk, NY), with *p* < 0.05 as a level of statistical significance.

## 3 RESULTS

### 3.1 ZZCD alleviates depression‐like behaviours and neuron injury in CUMS mice

**FIGURE 1:**
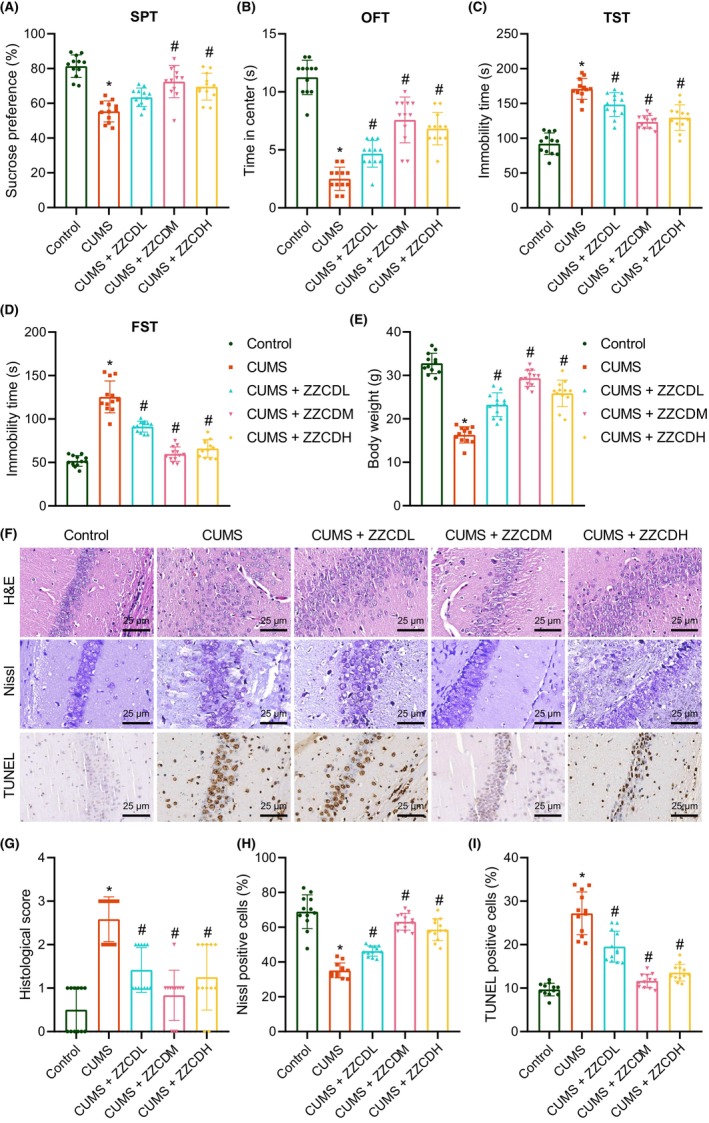
ZZCD ameliorates depression‐like behaviours and neuron injury in CUMS mice. (A) Sucrose preference of CUMS mice treated with low‐, medium‐ and high‐dose ZZCD tested by SPT. (B) Retention time in the open field centre of CUMS mice treated with low‐, medium‐ and high‐dose ZZCD tested by OFT. (C) Immobility time of tail suspension of CUMS mice treated with low‐, medium‐ and high‐dose ZZCD tested by TST. (D) Immobility time of forced swimming of CUMS mice treated with low‐, medium‐ and high‐dose ZZCD tested by FST. (E) Body weight of CUMS mice treated with low‐, medium‐ and high‐dose ZZCD. (F) Neuron injury in hippocampal tissues of CUMS mice treated with low‐, medium‐ and high‐dose ZZCD analysed by HE, Nissl and TUNEL staining. (G) Statistical analysis of pathological scores of hippocampal tissues of CUMS mice treated with low‐, medium‐ and high‐dose ZZCD. (H) Statistical analysis of Nissl‐positive neurons in hippocampal tissues of CUMS mice treated with low‐, medium‐ and high‐dose ZZCD. (I) Statistical analysis of TUNEL‐positive neurons in the hippocampal tissue of CUMS mice treated with low‐, medium‐ and high‐dose ZZCD. *n* = 12 mice for each treatment. *p < 0.05 compared with control mice, #*p* < 0.05 compared with untreated CUMS mice.

We first created CUMS models for mimicking depression symptoms in mice and then conducted behavioural tests. The results showed that CUMS mice had decreased sucrose preference and retention time in the open field centre yet increased immobility time of tail suspension and forced swimming compared with control mice. However, ZZCD treatment reversed these results (Figure 1A–D). In addition, body weight of CUMS mice was decreased compared with control mice while it was increased in the presence of ZZCD (Figure 1E). These results demonstrated the successful establishment of the CUMS‐induced depression mouse model and that ZZCD attenuated CUMS‐induced depression‐like behaviours in mice.

As shown in Figure 1F–I, HE staining results showed that hippocampal pyramidal cells of control mice were densely, tightly and regularly arranged, with intact cellular structure and clear edges. Conversely, cells of CUMS mice showed irregular and loose arrangement, accompanied by oedema and degeneration while ZZCD treatment restored the hippocampal pyramidal cell layer and reduced cellular oedema and degeneration. Besides, Nissl staining results revealed that neurons in the hippocampal CA1 region of control mice were compactly arranged and Nissl bodies were clearly visible while those in CUMS mice were irregular and sparsely distributed, with disintegrated Nissl bodies, and reduced Nissl‐positive neurons. ZZCD treatment attenuated the decrease of Nissl‐positive neurons and neuron injury. In addition, neuron apoptosis was augmented in the hippocampal tissue of CUMS mice but it was suppressed upon ZZCD treatment. The medium‐dose ZZCD (5 g/kg) had the most obvious alleviation effect on depressive‐like behaviours and neuron damage in CUMS mice. Therefore, we chose this dose for further animal experiments. These results demonstrated that ZZCD could relieve depression‐like behaviours and neuron injury in CUMS mice.

### 3.2 Network pharmacology and bioinformatics analysis identify BDNF as a key target for the treatment of depression

**FIGURE 2:**
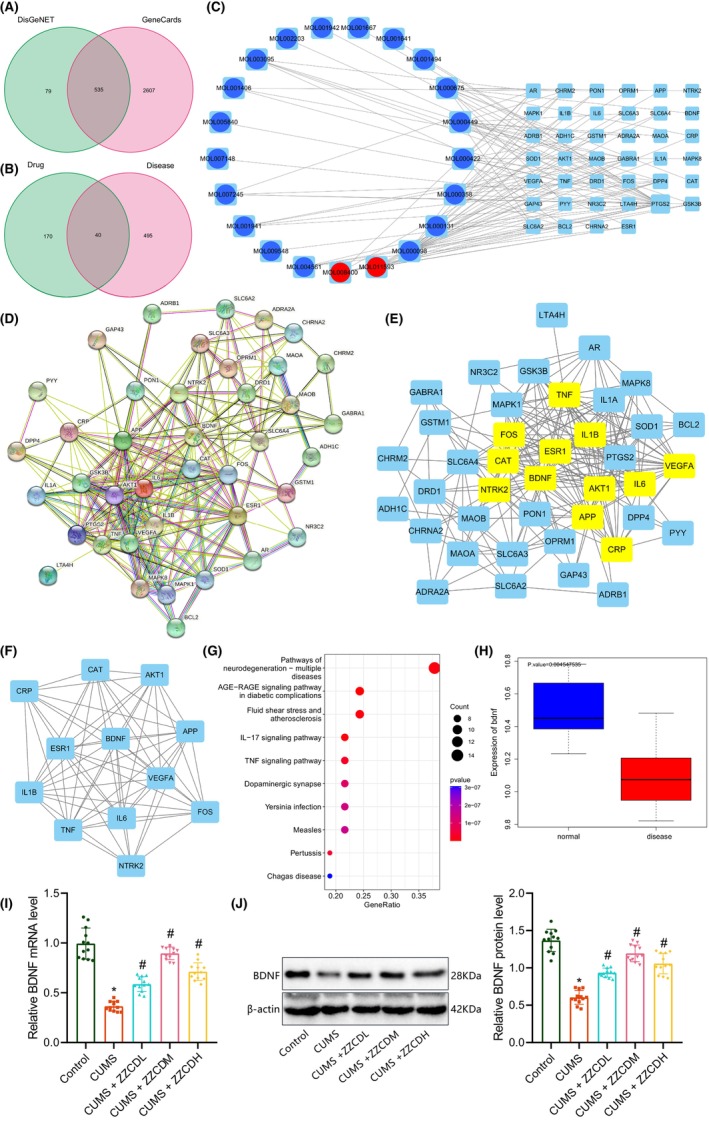
Network pharmacology and bioinformatics analysis of BDNF significance in ZZCD treating depression. (A) Venn diagram of the target genes related to depression retrieved from the GeneCards and DisGeNET databases. (B) Venn diagram of the depression‐related target genes with the target genes of active ingredients. (C) A network diagram of active ingredients and target genes. The left circle is the active ingredients of ZZ (blue) and DDC (red), and the right node is the target gene. (D) PPI network of the 40 candidate target genes constructed by STRING database. Different colour nodes represent proteins encoded by genes, and the connection between nodes represents the interaction between proteins. (E) Interaction network of 40 candidate target genes drawn by Cytoscape software. The yellow gene represents the candidate target gene filtered by the CytoNCA plug‐in. (F) Interaction network of 12 candidate target genes filtered by CytoNCA plug‐in. (G) KEGG pathway enrichment analysis of 40 candidate target genes. The abscissa represents Gene ratio, and different colours represent the *p* value, with redder colour reflecting lower *p* values and more significant difference. The dot size indicates the number of entry identifiers of genes, with larger dot representing more entry identifiers. (H) A box plot of the differential expression of BDNF gene in eight normal samples and eight disease samples in the GSE84183 dataset. (I) BDNF expression in the hippocampal tissue of CUMS mice and those treated with low‐, medium‐ and high‐dose ZZCD examined by RT‐qPCR. (J) Western blot of BDNF protein in the hippocampal tissue of CUMS mice and those treated with low‐, medium‐ and high‐dose ZZCD. *n* = 12 mice for each treatment. *p < 0.05 compared with control mice, #*p* < 0.05 compared with untreated CUMS mice.

To explore the main targets of ZZCD in treating depression, we searched the main active ingredients of ZZ and DDC in ZZCD from the TCMSP database and screened them with OB >30% and DL >0.1 as the threshold. A total of 26 and 3 active ingredients were obtained, respectively (Table S5). The targets of these active ingredients were further retrieved in the TCMSP database, with 210 target genes obtained (Table S6). Following Venn diagram analysis of the target genes related to depression retrieved from the GeneCards and DisGeNET databases, 535 genes were identified (Figure 2A) and then intersected with the above target genes of active ingredients, with 40 candidate target genes yielded (Figure 2B). A network map of the active ingredients and target genes is shown in Figure 2C, and an PPI network of the 40 target genes is displayed in Figure 2D. After scoring and filtering of each node gene by the CytoNCA plug‐in, 12 key target genes were obtained, including APP, AKT1, CAT, CRP, IL1B, FOS, IL6, TNF, ESR1, BDNF, VEGFA and NTRK2 (Figure 2E,F). Of them, the core degree of each score of BDNF gene (Betweenness, Closeness, Degree, Eigenvector and Network) was the highest (Table S7).

KEGG pathway enrichment analysis suggested that the 40 candidate target genes were mainly enriched in pathways of neurodegeneration‐multiple diseases, AGE‐RAGE signalling pathway in diabetic complications and Fluid shear stress and atherosclerosis, and BDNF was also significantly enriched in the pathways of neurodegeneration‐multiple diseases (Figure 2G).

Analysis of the GSE84183 dataset indicated low BDNF expression in the hippocampal tissue samples of mice with depression‐like behaviours (Figure 2H). Meanwhile, RT‐qPCR and western blot validated this low expression in the hippocampal tissue of CUMS mice while ZZCD treatment increased BDNF expression (Figure 2I,J). The above results suggest that BDNF may be a key target for ZZCD in the treatment of depression.

### 3.3 ZZCD attenuates CORT‐induced hippocampal neuron injury and upregulates BDNF expression

**FIGURE 3:**
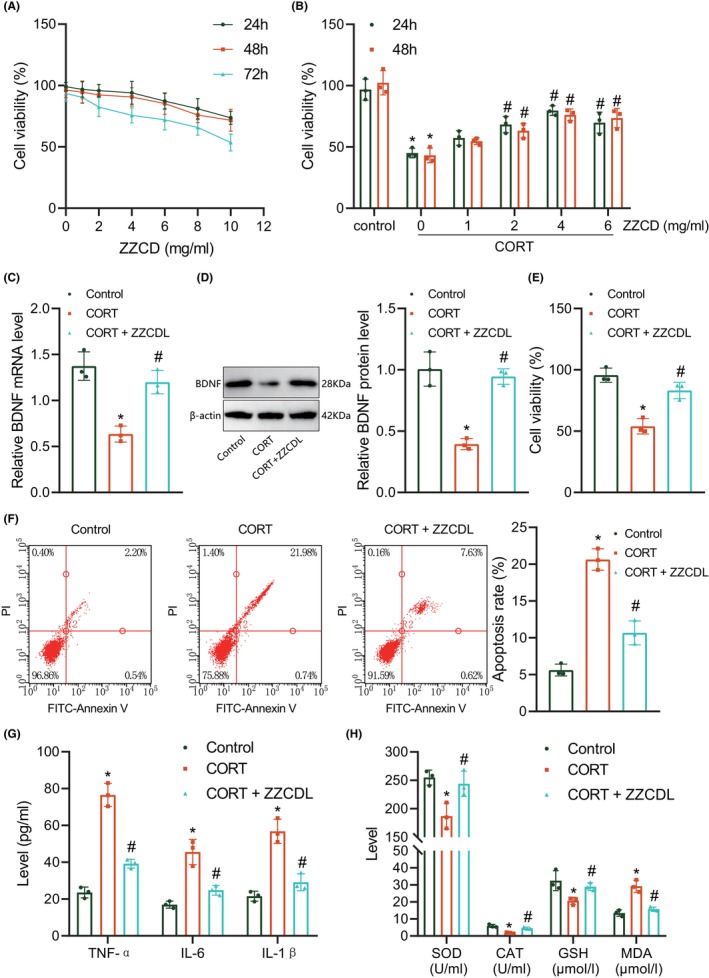
ZZCD represses CORT‐induced hippocampal neuron injury and upregulates BDNF expression. (A) Viability of hippocampal neurons treated with ZZCD at varied concentrations detected by CCK‐8 assay. (B) Viability of CORT‐stimulated hippocampal neurons treated with ZZCD at varied concentrations detected by CCK‐8 assay. (C) BDNF expression in CORT‐stimulated hippocampal neurons treated with ZZCD detected by RT‐qPCR. (D) Western blot of BDNF protein in the CORT‐stimulated hippocampal neurons treated with ZZCD. (E) Viability of CORT‐stimulated hippocampal neurons treated with ZZCD detected by CCK‐8 assay. (F) Apoptosis of CORT‐stimulated hippocampal neurons treated with ZZCD detected by flow cytometry. (G) Levels of TNF‐α, IL‐1β and IL‐6 in the supernatant of CORT‐stimulated hippocampal neurons treated with ZZCD detected by ELISA. (H), SOD, GSH and CAT activities and MDA production in the supernatant of CORT‐stimulated hippocampal neurons treated with ZZCD detected by ELISA. *p < 0.05 compared with control neurons, #*p* < 0.05 compared with untreated CORT‐stimulated neurons. The cell experiment was repeated three times.

Then, to evaluate the effect of ZZCD on neuron injury following depression in vitro, we first isolated primary hippocampal neurons from mice and identified them by immunofluorescence. More than 95% of MAP‐2‐positive cells were present in neurons (Figure S2A). CCK‐8 assay and flow cytometry results showed a decline in the viability of CORT‐stimulated neurons and an increase in cell apoptosis relative to control cells (Figure S2B,C). In Figure 3A, 0–6 mg/mL ZZCD did not affect hippocampal neuron viability at 24–48 h, but ZZCD >6 mg/mL inhibited cell viability. In addition, ZZCD at varied concentrations showed inhibitory roles in cell viability at 72 h. Besides, treatment with 4 mg/mL ZZCD at 24 h reversed the inhibition of CORT on cell viability (Figure 3B). Therefore, we selected 4 mg/mL ZZCD treatment for 24 h for the subsequent experiments.

Based on the results of RT‐qPCR and western blot, BDNF expression was decreased in the CORT‐stimulated cells while an increase was noted in the presence of ZZCD (Figure 3C,D). Cell viability was suppressed and cell apoptosis was potentiated following CORT stimulation, the effect of which was negated by ZZCD (Figure 3E,F). ELISA data exhibited the levels of TNF‐α, IL‐1β and IL‐6 as well as MDA production were increased in the supernatant of the CORT‐stimulated cells while SOD, GSH and CAT activities were reduced. ZZCD reduced inflammatory response and oxidative stress damage (Figure 3G,H). The above results suggest that ZZCD could alleviate CORT‐induced hippocampal neuron injury and upregulate BDNF expression.

### 3.4 Six3os1 recruits KMT2A to the BDNF promoter and promotes histone H3K4me3, thus elevating BDNF expression

**FIGURE 4:**
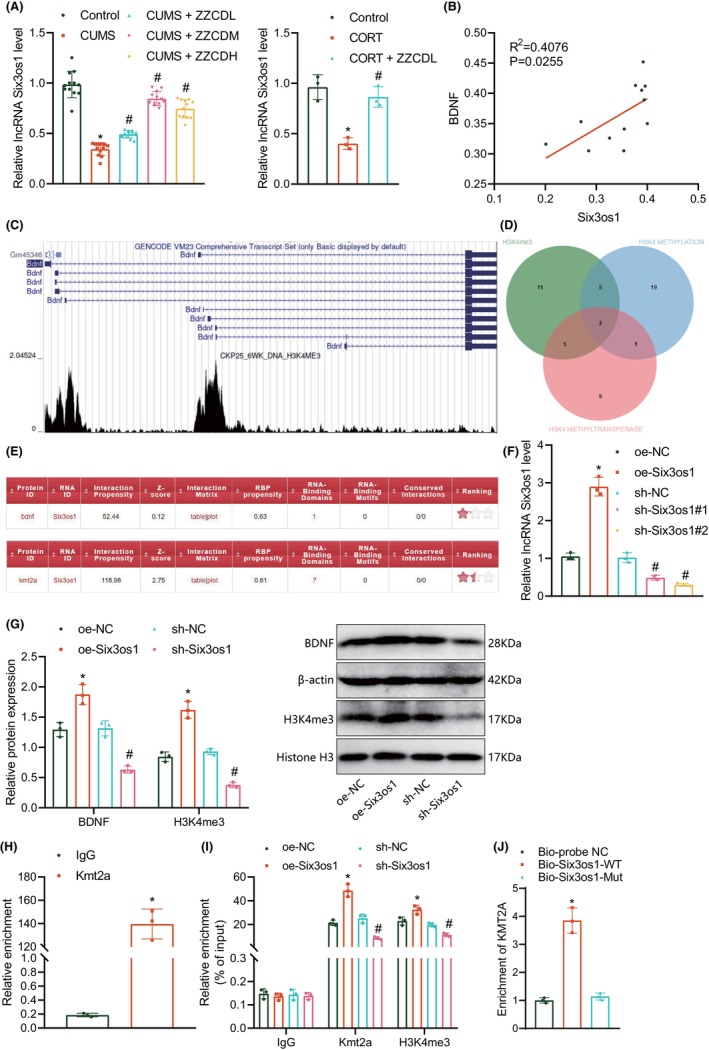
Six3os1 increases BDNF expression by recruiting KMT2A to the BDNF promoter and promoting histone H3K4me3. (A) Six3os1 expression in the hippocampal tissue of control and CUMS mice detected by RT‐qPCR. *p < 0.05 compared with control neurons, # *p* < 0.05 compared with untreated CORT‐stimulated neurons, (B) Correlation analysis of Six3os1 and BDNF expression, with 12 mice in each group. (C) H3K4me3 modification in the BDNF promoter region analysed by UCSC database. (D) Venn diagram of genes encoding H3K4me3‐associated transferases in the GSEA‐MSigDB database. (E) The binding between KMT2A and Six3os1 predicted by catRAPID website. (F) Six3os1 expression in primary hippocampal neurons treated with oe‐Six3os1 or sh‐Six3os1 detected by RT‐qPCR. *p < 0.05 compared with neurons transduced with oe‐NC, #*p* < 0.05 compared with neurons transduced with sh‐NC. (G) Western blot of BDNF and H3K4me3 proteins in primary hippocampal neurons treated with sh‐Six3os1.*p < 0.05 compared with neurons transduced with oe‐NC, #*p* < 0.05 compared with neurons transduced with sh‐NC. (H) RIP assay of Six3os1 binding to KMT2A protein in primary hippocampal neurons. *p < 0.05 compared with IgG. (I) ChIP assay of KMT2A and H3K4me3 enrichment in the BDNF promoter region in primary hippocampal neurons. (J) RNA pull‐down experiment to detect the binding of Six3os1 and KMT2A promoter in primary hippocampal neurons. *p < 0.05 compared with neurons transduced with oe‐NC or Bio‐probe‐NC, #*p* < 0.05 compared with neurons transduced with sh‐NC. The cell experiment was repeated three times.

Six3os1 expression in the hippocampal tissue of CUMS mice and CORT‐stimulated hippocampal neurons was detected by RT‐qPCR, which revealed a decline of Six3os1 expression in the two models, while ZZCD abolished this decline (Figure 4A). This indicates that Six3os1 expression was low in the context of depression and could be upregulated by ZZCD. In addition, a positive correlation was detected between Six3os1 expression and BDNF expression in the hippocampal tissue of CUMS mice (Figure 4B). Accordingly, we hypothesized that Six3os1 may regulate BDNF and play an important role in ZZCD treating depression.

H3K4me3 modification sites were found in the BDNF promoter region (Figure 4C). Through the GSEA‐MSigDB database, two candidate H3K4 methyltransferase genes were retrieved, namely KMT2A and PRDM9 (Figure 4D). In addition, the catRAPID website predicted the binding between KMT2A and Six3os1 (Figure 4E). Six3os1 was predicted to be mainly localized in the nucleus by the lncLocator website and FISH confirmed that Six3os1 was mainly localized in the nucleus of hippocampal neurons (Figure S3). Therefore, we hypothesized that Six3os1 may recruit KMT2A and mediate histone H3K4 methylation of the BDNF promoter, thus regulating BDNF expression.

RT‐qPCR results manifested increased Six3os1 expression in the primary hippocampal neurons transfected with oe‐Six3os1 and decreased Six3os1 expression in the presence of sh‐Six3os1#1 or sh‐Six3os1#2. sh‐Six3os1#2 showed the better efficiency and was used for subsequent assays (Figure 4F). Protein expression of BDNF and H3K4me3 was elevated in hippocampal neurons overexpressing Six3os1 whereas opposite results were found upon Six3os1 silencing (Figure 4G). The enrichment of Six3os1 was increased in cell lysates incubated with anti‐KMT2A antibody (Figure 4H). In addition, the enrichment of KMT2A and H3K4me3 in the BDNF promoter region was augmented in the presence of Six3os1 overexpression but this augmentation was inhibited upon Six3os1 silencing (Figure 4I). The RNA pull‐down results indicate a significant increase in the expression of the Kmt2a promoter enriched by Bio‐Six3os1‐Wt compared to Bio‐probe‐NC and io‐Six3os1‐Mut (Figure 4J). These data highlight that Six3os1 may promote the histone H3K4 methylation of the BDNF promoter possibly by recruiting KMT2A. Cumulatively, Six3os1 may upregulate the expression of BDNF by recruiting KMT2A to the BDNF promoter region to promote histone H3K4me3.

### 3.5 Six3os1 upregulates BDNF expression to attenuate CORT‐induced hippocampal neuron injury

**FIGURE 5:**
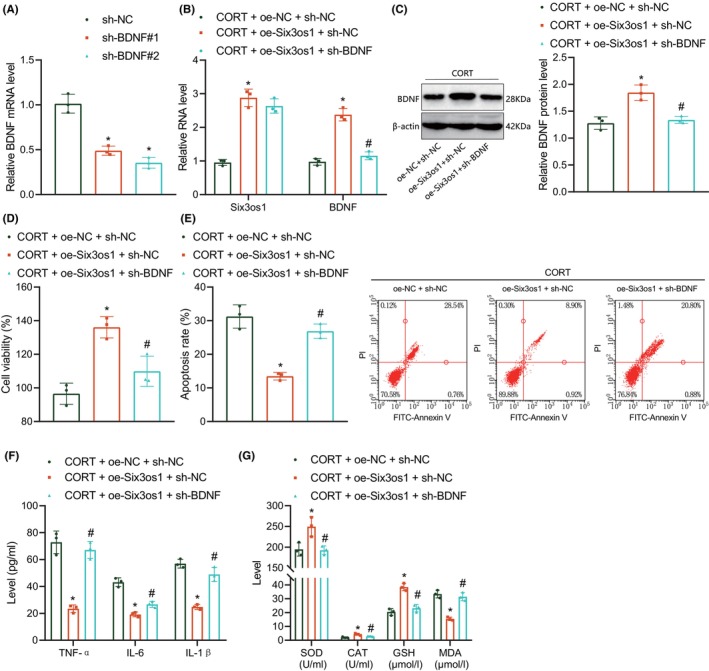
Six3os1 elevates BDNF expression and alleviates CORT‐induced hippocampal neuron injury. (A) BDNF knockdown efficiency in hippocampal neurons detected by RT‐qPCR. *p < 0.05 compared with neurons transduced with sh‐NC. (B) Six3os1 and BDNF expression in CORT‐stimulated hippocampal neurons treated with oe‐Six3os1 or combined with sh‐BDNF detected by RT‐qPCR. (C) Western blot of BDNF protein in CORT‐stimulated hippocampal neurons treated with oe‐Six3os1 or combined with sh‐BDNF. (D) CCK‐8 assay of cell viability of CORT‐stimulated hippocampal neurons treated with oe‐Six3os1 or combined with sh‐BDNF. (E) Apoptosis of CORT‐stimulated hippocampal neurons treated with oe‐Six3os1 or combined with sh‐BDNF measured by flow cytometry. (F) Levels of TNF‐α, IL‐1β and IL‐6 in the supernatant of CORT‐stimulated hippocampal neurons treated with oe‐Six3os1 or combined with sh‐BDNF detected by ELISA. (G) SOD, GSH and CAT activities and MDA production in the supernatant of CORT‐stimulated hippocampal neurons treated with oe‐Six3os1 or combined with sh‐BDNF detected by ELISA. *p < 0.05 compared with CORT‐stimulated hippocampal neurons treated with oe‐NC + sh‐NC, #*p* < 0.05 compared with neurons transduced with CORT‐stimulated hippocampal neurons treated with oe‐Six3os1 + sh‐NC. The cell experiment was repeated three times.

Next, we aimed to verify the effect of Six3os1 on hippocampal neuron functions by regulating BDNF. RT‐qPCR results showed that BDNF expression was reduced in hippocampal neurons treated with sh‐BDNF#1 or sh‐BDNF#2, with sh‐BDNF#1 exhibiting the superior knockdown efficiency and selected for subsequent experiments (Figure 5A). The expression of Six3os1 and BDNF was noted to upregulated upon oe‐Six3os1 treatment while further silencing of BDNF diminished BDNF expression without altering Six3os1 expression (Figure 5B,C).

As revealed by CCK‐8 and flow cytometry, Six3os1 overexpression potentiated hippocampal neuron viability and decreased cell apoptosis, which were rescued by further silencing of BDNF (Figure 5D,E). Moreover, ELISA data showed a reduction in the levels of TNF‐α, IL‐1β and IL‐6 as well as MDA production yet an enhancement in the activities of SOD, GSH and CAT upon Six3os1 overexpression, the effect of which was undermined by BDNF silencing (Figure 5F,G). Overall, Six3os1 can reduce CORT‐induced hippocampal neuron injury by upregulating BDNF expression.

### 3.6 ZZCD reduces CORT‐induced hippocampal neuron injury by upregulating the Six3os1/BDNF axis in vitro

**FIGURE 6:**
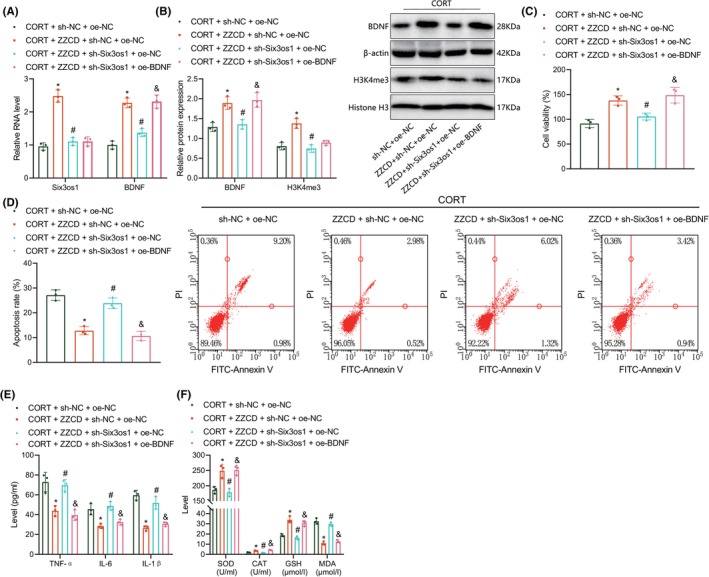
ZZCD represses CORT‐induced hippocampal neuron injury by upregulating the Six3os1/BDNF axis. CORT‐stimulated hippocampal neurons were treated with ZZCD + sh‐NC + oe‐NC, ZZCD + sh‐Six3os1 + oe‐NC or ZZCD + sh‐Six3os1 + oe‐BDNF. (A) Expression of Six3os1 and BDNF in hippocampal neurons detected by RT‐qPCR. (B) Western blot of BDNF and H3K4me3 proteins in hippocampal neurons. (C) Viability of hippocampal neurons detected by CCK‐8 assay. (D) Apoptosis of hippocampal neurons detected by flow cytometry. (E) Levels of TNF‐α, IL‐1β and IL‐6 in the supernatant of hippocampal neurons detected by ELISA. (F) SOD, GSH and CAT activities and MDA production in the supernatant of hippocampal neurons detected by ELISA. *p < 0.05 compared with CORT‐stimulated hippocampal neurons treated with oe‐NC + sh‐NC, #*p* < 0.05 compared with CORT‐stimulated hippocampal neurons treated with ZZCD + oe‐NC + sh‐NC, &*p* < 0.05 compared with CORT‐stimulated hippocampal neurons treated with. ZZCD + oe‐Six3os1 + sh‐NC. The cell experiment was repeated three times.

The aforementioned results inspired us to examine whether ZZCD suppressed CORT‐induced hippocampal neuron injury by regulating the Six3os1/BDNF axis. RT‐qPCR and western blot results demonstrated that the increased expression of Six3os1, H3K4me3 and BDNF in CORT‐stimulated hippocampal neurons following treatment with ZZCD was abrogated by further treatment with sh‐Six3os1. In contrast, treatment with ZZCD + sh‐Six3os1 + oe‐BDNF restored BDNF expression without affecting Six3os1 and H3K4me3 expression (Figure 6A,B). These results showed that ZZCD can promote the modification of H3K4me3 in the promoter region of BDNF by upregulating Six3os1, thereby increasing BDNF expression.

Moreover, augmented cell viability, increased SOD, GSH and CAT activities, and reduced cell apoptosis, levels of TNF‐α, IL‐1β and IL‐6 and MDA production due to ZZCD were abolished by Six3os1 silencing. However, ectopically expressed BDNF restored the effect of ZZCD on these indicators (Figure 6C–F). These lines of evidence unveil that ZZCD could reduce CORT‐induced hippocampal neuron injury by upregulating Six3os1 and BDNF expression.

### 3.7 ZZCD reduces depression‐like behaviours and neuron injury by upregulating the Six3os1/BDNF axis in vivo

**FIGURE 7:**
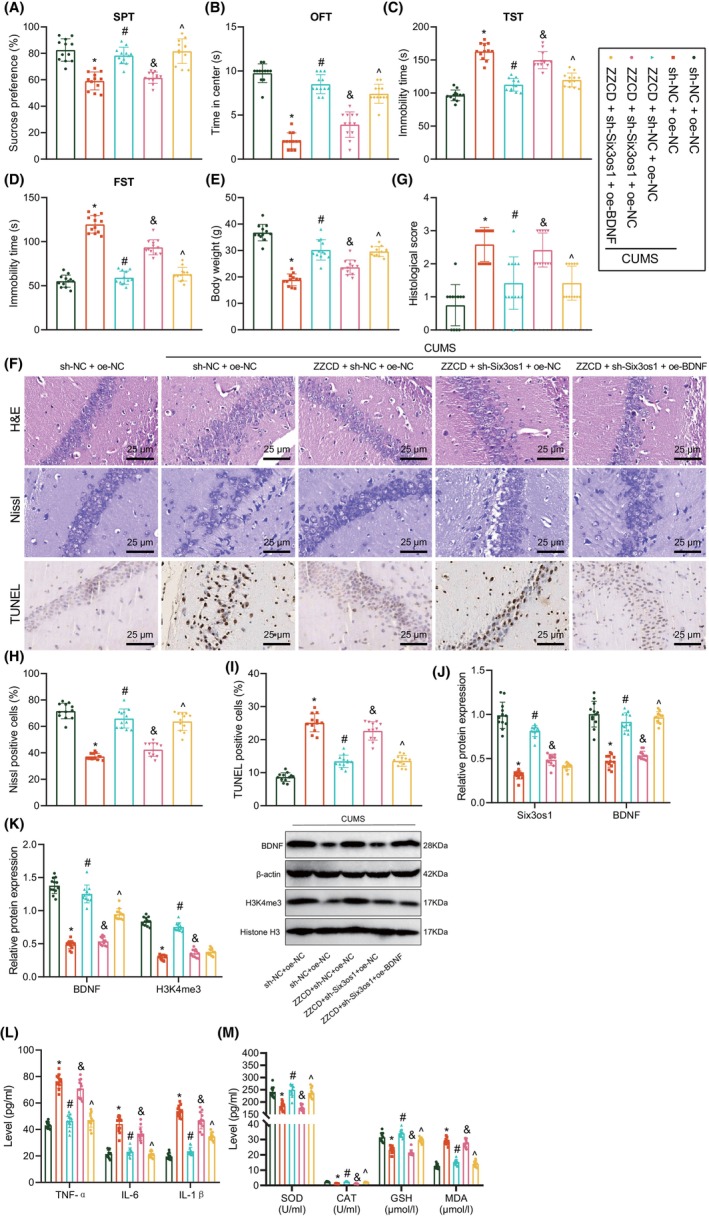
ZZCD alleviates depression‐like behaviours and neuron injury in mice by upregulating the Six3os1/BDNF axis. CUMS mice were treated with ZZCD + sh‐NC + oe‐NC, ZZCD + sh‐Six3os1 + oe‐NC or ZZCD + sh‐Six3os1 + oe‐BDNF. (A) Sucrose preference of CUMS mice tested by SPT. (B) Retention time in the open field centre of CUMS mice tested by OFT. (C) Immobility time of tail suspension of CUMS mice tested by TST. (D) Immobility time of forced swimming of CUMS mice tested by FST. (E) Body weight of CUMS mice. (F) Neuron injury in hippocampal tissues of CUMS mice analysed by HE, Nissl and TUNEL staining. (G) Statistical analysis of pathological scores of hippocampal tissues of CUMS mice. (H) Statistical analysis of Nissl‐positive neurons in hippocampal tissues of CUMS mice. (I) Statistical analysis of TUNEL‐positive neurons in the hippocampal tissue of CUMS mice. (J) Expression of Six3os1 and BDNF in hippocampal tissues of CUMS mice detected by RT‐qPCR. (K) Western blot of BDNF and H3K4me3 proteins in hippocampal tissues of CUMS mice. (L) Levels of TNF‐α, IL‐1β and IL‐6 in mouse serum detected by ELISA. (M) SOD, GSH and CAT activities and MDA production in mouse serum detected by ELISA. *n* = 12 mice for each treatment. *p < 0.05 compared with control mice treated with oe‐NC + sh‐NC, #*p* < 0.05 compared with CUMS mice treated with oe‐NC + sh‐NC, &*p* < 0.05 compared with CUMS mice treated with ZZCD + oe‐NC + sh‐NC, ^*p* < 0.05 compared with CUMS mice treated with. ZZCD + oe‐Six3os1 + sh‐NC.

Finally, we aimed to characterize the mechanism of antidepressant effects of ZZCD by regulating the Six3os1/BDNF axis. The behavioural test results displayed that Six3os1 silencing counterweighed the antidepressant effects of ZZCD, as evidenced by reduced sucrose preference and retention time in the open field centre yet increased immobility time of tail suspension and forced swimming. Conversely, ectopically expressed BDNF restored the antidepressant effects of ZZCD (Figure 7A–D). In addition, ZZCD‐induced increased body weight of CUMS mice was counteracted in the presence of Six3os1 silencing but additional BDNF overexpression increased body weight (Figure 7E). These results demonstrated that ZZCD attenuated CUMS‐induced depression‐like behaviours by upregulating the Six3os1/BDNF axis in mice.

Additionally, inhibited cell injury and apoptosis in the hippocampal tissue of CUMS mice in response to ZZCD treatment were undermined by Six3os1 silencing whereas further BDNF overexpression reduced cell injury and apoptosis (Figure 7F–I).

As demonstrated by RT‐qPCR and western blot, upregulated Six3os1, H3K4me3 and BDNF expression induced by ZZCD was inhibited by sh‐Six3os1, and conversely, oe‐BDNF elevated BDNF expression (Figure 7J,K). Furthermore, ELISA data presented that Six3os1 silencing reversed the inhibiting effect of ZZCD on the inflammatory response and oxidative stress injury. However, ectopically expressed BDNF reduced inflammatory response and oxidative stress injury (Figure 7L,M). Altogether, ZZCD can prevent depression‐like behaviours and neuron injury in mice by upregulating Six3os1 and BDNF expression.

## 4 DISCUSSION

TCM offers a unique and holistic approach to understanding and treating depression.^32^ Additionally, TCM can offer a complementary mode of treatment for individuals who have not responded well to conventional therapies or experience adverse effects from medications.^33^ ZZCD has been used as a drug therapy for depression due to its effects and limited side effects.^34^ Accordingly, this study was aimed at investigating the mechanism of ZZCD in depression. Through an array of experiments, the present study illustrated that ZZCD alleviated the oxidative stress and neuron injury as well as the resultant depression through activation of the Six3os1/BDNF axis.

In TCM, depression is viewed as an imbalance of Qi and organ systems that can lead to disruptions in physical and emotional health.^35^ Results of the present study revealed that ZZCD could ameliorate depression‐like behaviours in CUMS mice. Indeed, previous evidence has highlighted that ZZCD has a positive treatment effect on CUMS‐induced depression models in mice as the reduced mouse body weight and sucrose preference yet increased immobility time, total distances and time in centre induced by CUMS can be reversed by high‐ and medium‐dose ZZCD.^36^ Meanwhile, a recent study has identified the protective effect of ZZCD on PC12 cells against oxidative stress and apoptosis.^37^ This finding concurs with ours that ZZCD can attenuate CORT‐induced hippocampal neuron injury.

BDNF is a protein that promotes the growth and survival of neurons in the brain and is crucial in maintaining proper brain function.^38^ Research has linked BDNF to depression, and TCM has been shown to improve BDNF levels in individuals with depression.^39^ Multiple TCM modalities have been shown to improve BDNF levels, including herbal medicine, acupuncture, dietary therapy and mind–body practices such as tai chi.^40^ Subsequent network pharmacology and bioinformatics analysis together with experimental validation revealed that BDNF may be a key target for ZZCD in the treatment of depression. Consistently, ZZCD exhibits antidepressant effects by regulating gut microbiota to induce butyrate, which further regulates neurotransmitters, endocrine and BDNF along the gut‐brain axis.^41^ In addition, the anti‐depression efficacy of ZZCD may be associated with PKA‐CREB‐BDNF–TrkB‐PSD‐95 signalling pathway influenced by metabolic changes.^36^ Recent research has indicated that depression is a consequence of reduced BDNF expression in the brain and increasing BDNF expression through antidepressant therapy thus shows positive response in the treatment of depression.^42^ Besides, previously published literature has signified that BNDF aids in the protection of neurons from damage caused by infection or injury.^43^ Huang et al found that BDNF expression is decreased in hypoxic‐hypoglycaemic hippocampal neurons, and that abundant expression of BDNF increases hippocampal neuron survival and reduces cell apoptosis by inhibiting miR‐134 expression and activating the TrkB pathway.^44^

Another key observation of the current study indicated that Six3os1 could recruit KMT2A to the BDNF promoter and promotes histone H3K4me3, thus elevating BDNF expression. Six3os1‐mediated upregulation of BDNF consequently attenuated CORT‐induced hippocampal neuron injury. Six3os1/KMT2A is a biological pathway involved in the regulation of gene expression, with emerging data suggesting its involvement in the pathogenesis of psychiatric disorders such as depression.^1^ Evidence has shown that BDNF expression is influenced by the epigenetic modification at its promoter region; specifically, high BDNF methylation can reduce mRNA expression of BDNF.^23^ BHBA is capable of enhancing the expression of BDNF by increasing H3K4me3 in hippocampal neurons.^45^ In addition, repressive histone modifications at the BDNF promoter in the hippocampus of rats can reduce transcriptional capacity of the gene encoding BDNF.^46^ Notably, BDNF promoter methylation might be a biomarker for depression as high BDNF methylation links to the incidence of depression and severe depressive symptoms.^47^ KMT2A has been involved in histone modifications.^48^ The binding of KMT2A to the BDNF promoter region 2 is increased by chronic morphine, accompanied by enhancement in the H3K4me3 levels.^20^ Intriguingly, Geniposide, an iridoid glycoside derived from *Fructus Gardeniae*, can upregulate the expression of Six3os1 in CUMS‐induced neurons and this upregulation is capable of disrupting oxidative stress in CUMS‐induced mice and neurons, and suppressing CUMS‐induced neuron apoptosis.^17^ Considering the aforementioned evidence, it can be concluded that ZZCD reduces CORT‐induced hippocampal neuron injury in vitro and reduces depression‐like behaviours and neuron injury in vivo by upregulating the Six3os1/BDNF axis. These findings may help to identify a potential therapeutic agent for the treatment of depression.

Altogether, the key findings obtained from this study suggest the potential effect of ZZCD treatment on restricting oxidative stress and neuron injury following depression by upregulating Six3os1 and BDNF expression (Figure S4). The functional mechanism of ZZCD via the Six3os1/BDNF axis unveiled in the current study provides novel therapeutic hints for depression prevention and treatment. However, it should also be noted whether the therapeutic target is applicable to human beings requires further verification. Additional studies have also suggested detecting the interaction between Six3os1 and BDNF to further confirm that Six3os1 upregulates BDNF expression by mediating the histone H3K4 methylation modification of the BDNF promoter. The elucidated mechanism of ZZCD through Six3os1/KMT2A/H3K4me3 pathway and the subsequent stimulation of BDNF expression can lead to targeted and effective treatments for depression, which can be further investigated and developed. Additionally, ZZCD's ability to alleviate CORT‐induced hippocampal neuron injury, inflammatory response and oxidative stress provides a potentially important therapeutic effect in depression. As with any research, there are limitations such as the lack of investigation of potential side effects and safety concerns; however, these results represent a significant step towards developing new and effective treatments for depression.

## AUTHOR CONTRIBUTIONS

**Tianyu Zou:** Conceptualization (equal); data curation (equal); funding acquisition (equal); writing – original draft (equal). **Kazuo Sugimoto:** Formal analysis (equal); investigation (equal); writing – original draft (equal). **Yu Zhao:** Investigation (equal); methodology (equal); writing – original draft (equal). **Baitao Li:** Resources (equal); software (equal); writing – review and editing (equal). **Xiaomao Zhou:** Supervision (equal); validation (equal); writing – review and editing (equal). **Cheng Peng:** Methodology (equal); visualization (equal); writing – review and editing (equal).

## FUNDING INFORMATION

This study was supported by Guangdong Basic and Applied Basic Research Foundation (No. 2021A1515110758 and 2019A1515011789), Medical Research Projects of Guangdong Province (A2024314), Shenzhen Science and Technology Program (No. JCYJ20230807150359008), Shenzhen Luohu District Soft Science (No. LX202202041 and LX202302095) and Shenzhen Health Elite Talent Training Program (No. 2022XKQ076).

## CONFLICT OF INTEREST STATEMENT

The authors declare no conflict of interest.

## Supporting information


Figure S1.

Figure S2.

Figure S3.

Figure S4.

Tables S1‐S4.

Table S5.

Table S6.

Table S7.

## Data Availability

The data underlying this article will be shared on reasonable request to the corresponding author.
