# Supplementary figures and images for: Zhi‐zi‐chi decoction mitigates depression by enhancing lncRNA Six3os1 expression and promoting histone H3K4 methylation at the BDNF promoter
Source: J Cell Mol Med. 2024 May 31;28(11):e18365. doi: 10.1111/jcmm.18365 (PMC11140235; doi:10.1111/jcmm.18365)

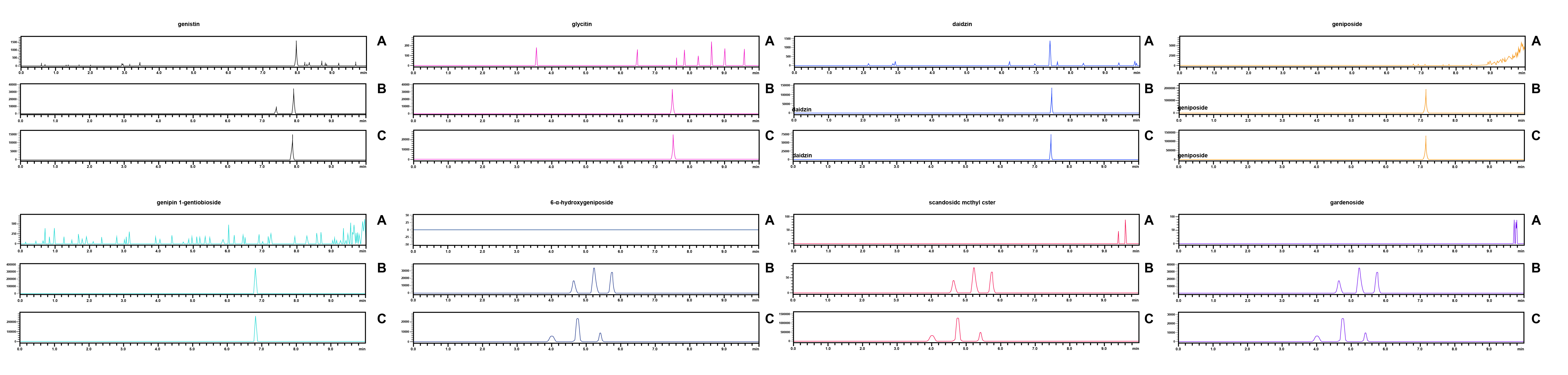

Supplement: Supplementary file 1 — Figure S1. [file JCMM-28-e18365-s007.jpg]

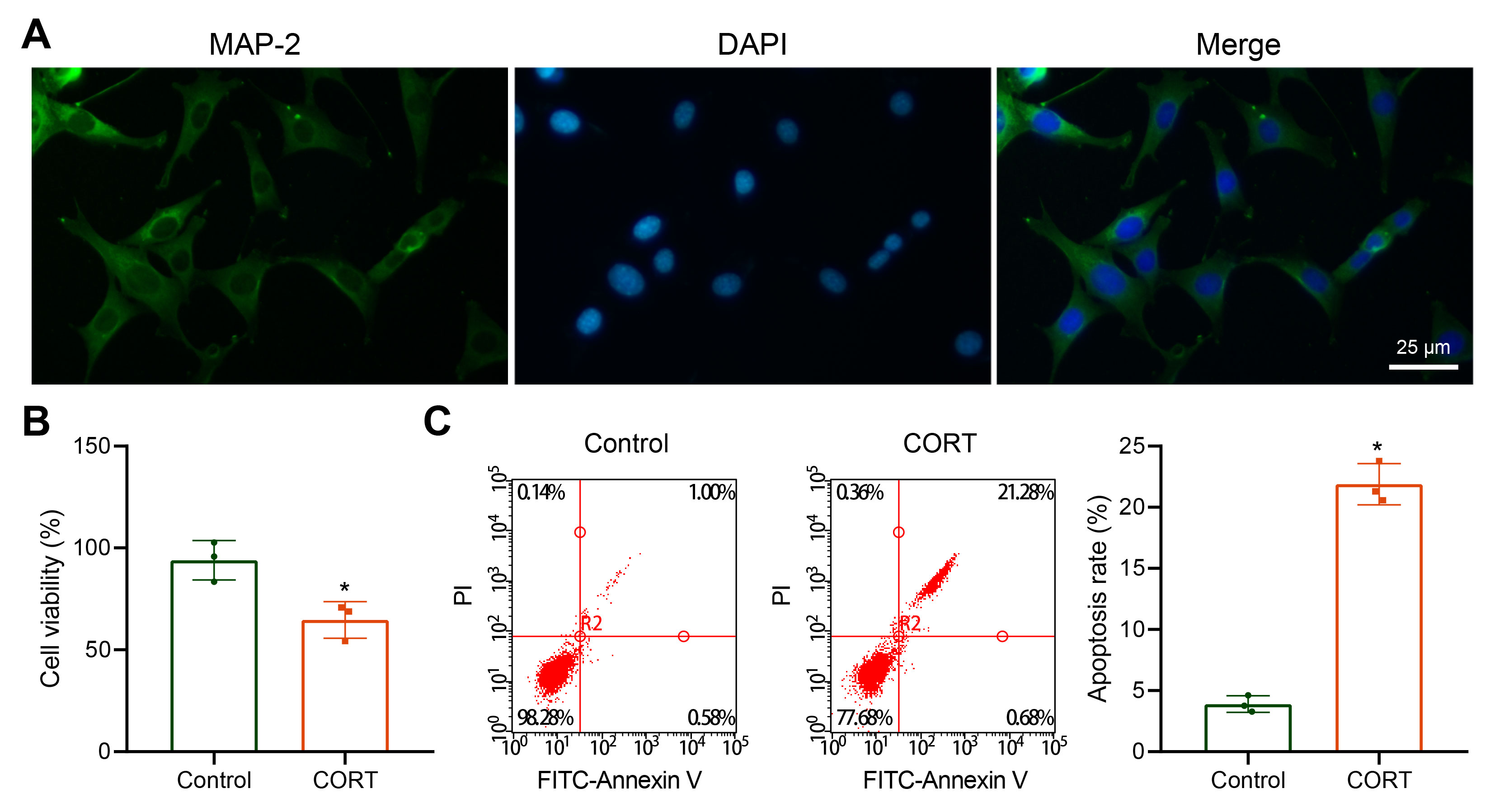

Supplement: Supplementary file 2 — Figure S2. [file JCMM-28-e18365-s003.jpg]

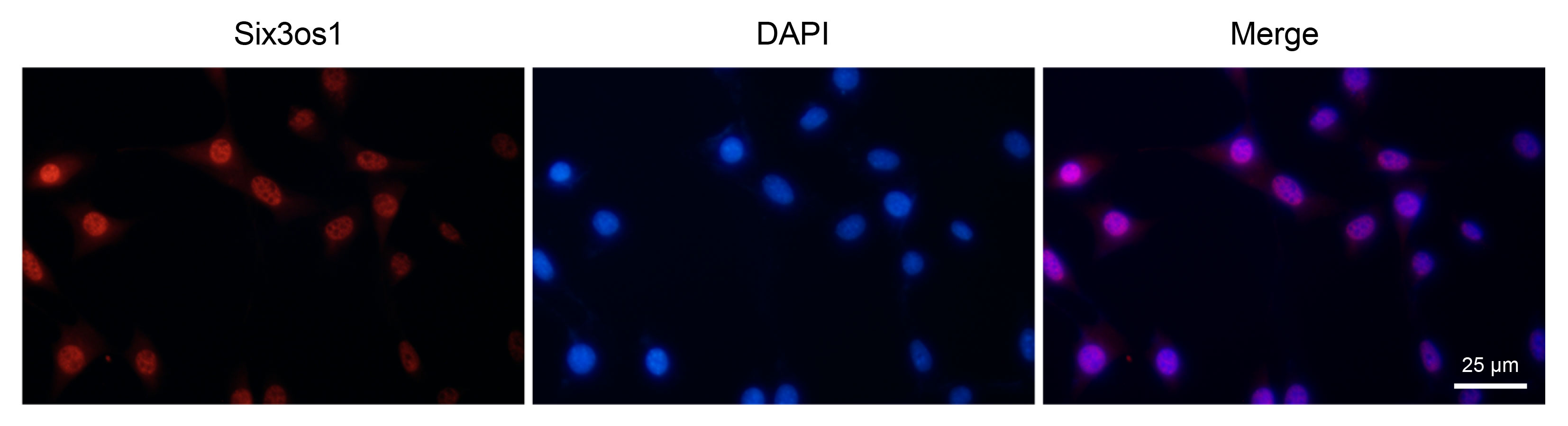

Supplement: Supplementary file 3 — Figure S3. [file JCMM-28-e18365-s008.jpg]

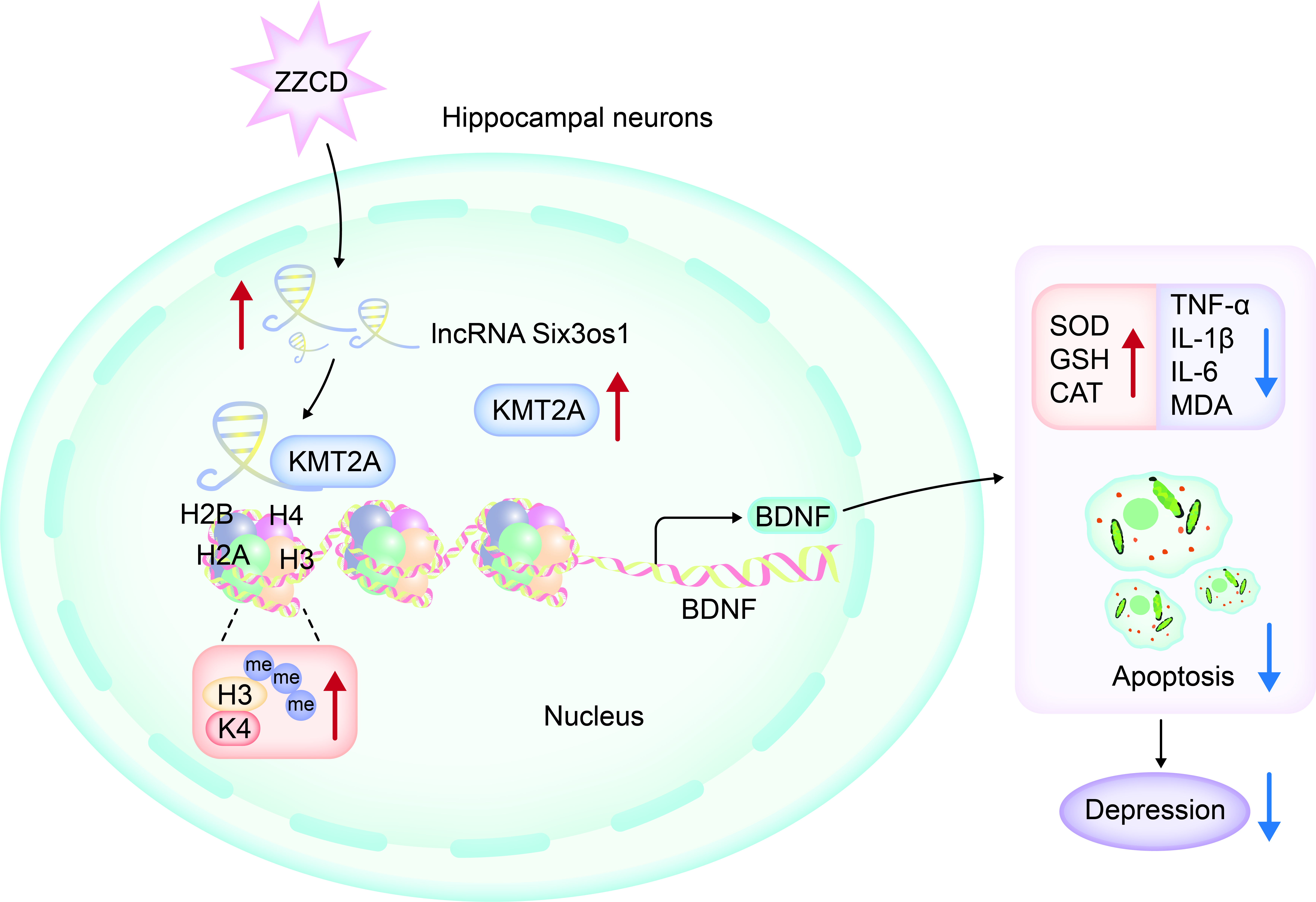

Supplement: Supplementary file 4 — Figure S4. [file JCMM-28-e18365-s005.jpg]
