# Supplementary material for: Zhi‐zi‐chi decoction mitigates depression by enhancing lncRNA Six3os1 expression and promoting histone H3K4 methylation at the BDNF promoter
Source: J Cell Mol Med. 2024 May 31;28(11):e18365. doi: 10.1111/jcmm.18365 (PMC11140235; doi:10.1111/jcmm.18365)
Supplement: Supplementary file 5 — Tables S1‐S4. [file JCMM-28-e18365-s002.docx]

**Supplementary Table 1: Quantitative Concentration Determination of 8 Components in ZZCD.**

| No | Compounds | Regression Equation of Calibration Curves | r | Linear Range (ng/mL) | Precisions (n = 6) RSD (%) | Reproducibility (n = 6) RSD (%) | Stability (n = 6) RSD (%) | Recovery (n = 3) Mean (%) | Recovery (n = 3) RSD (%) |
| --- | --- | --- | --- | --- | --- | --- | --- | --- | --- |
| 1 | genistin | A1 = 830.033C₁ - 1991.02 | 0.9995 | 5.0 - 100.0 | 0.66 | 1.8 | 1.27 | 102.63 | 1.78 |
| 2 | glycitin | A2 = 5945.29C₂ + 9532.52 | 0.9994 | 5.0 - 62.5 | 1.52 | 1.9 | 1.67 | 103.36 | 1.76 |
| 3 | daidzin | A3 = 3327.43C₃ + 21526.8 | 0.9994 | 5.0 - 100.0 | 1.67 | 1.7 | 1.58 | 102.23 | 1.4 |
| 4 | geniposide | A4 = 312.648C₄ + 321709 | 0.9994 | 200.0 - 25000.0 | 1.37 | 2 | 1.85 | 104.17 | 1.12 |
| 5 | genipin 1-gentiobioside | A5 = 235.414C₅ + 31245.7 | 0.9993 | 50.0 - 6250.0 | 1.18 | 1.8 | 1.62 | 103.9 | 2.94 |
| 6 | 6-α-hydroxygeniposide | A6 = 245.360C₆ - 60.2719 | 0.9998 | 50.0 - 62.5 | 1.71 | 1.7 | 1.72 | 103.53 | 1.32 |
| 7 | scaposide methyl ester | A7 = 559.336C₇ + 775.470 | 0.9995 | 20.0 - 250.0 | 1.23 | 1.9 | 1.61 | 102.45 | 1.26 |
| 8 | gardenoside | A8 = 382.451C₈ + 6115.09 | 0.9997 | 100.0 - 1250.0 | 1.62 | 1.3 | 1.48 | 102.45 | 1.26 |

**Supplementary Table 2 Details of CUMS-induced depression models in mice**

| No | Stress method | Description |
| --- | --- | --- |
| 1 | Damp bedding | Damp bedding for 24 h |
| 2 | Cage tilting | Cage tilting 45° for 12 h |
| 3 | Day-night reversal | Light/dark cycle reversal for 24 h |
| 4 | Food deprivation | Food deprivation for 24 h |
| 5 | Water deprivation | Water deprivation for 24 h |
| 6 | Lighting | Lighting for 12 h |
| 7 | Restraint | Forced physical restraint for 2 h |
| 8 | Tail clamping | Clamping the tail with tweezers for 10 min |
| 9 | Swimming | Swimming in 4°C cold water for 6 min |
| 10 | Oscillation | Oscillation for 5 min |

**Supplementary Table 3 shRNA sequences**

| shRNA | Sequence |
| --- | --- |
| sh-BDNF#1 | 5’-GCGCCCATGAAAGAAGTAAAC-3’ |
| sh-BDNF#2 | 5’-GGTGATGCTCAGCAGTCAAGT-3’ |
| sh-Six3os1#1 | 5’-GCAGCTAGCAGTTTGCTTTCG-3’ |
| sh-Six3os1#2 | 5’-GGACTAATACTCGTCCCTTAA-3’ |
| sh-NC | 5’-GCTTTGTGATTCAATCTGTAA-3’ |

**Supplementary Table 4 The primer sequence for RT-qPCR**

| Gene | Sequence (5’-3’) |
| --- | --- |
| Six3os1 | Forward: 5’-AGCAGTTTGCTTTCGCTCCT-3’ |
|  | Reverse: 5’-CATCGCAGGGCACCAACTAT-3’ |
| BDNF | Forward: 5’-GACGACATCACTGGCTGACA-3’ |
|  | Reverse: 5’-CAAGTCCGCGTCCTTATGGT-3’ |
| GAPDH | Forward: 5’-GGTCCCAGCTTAGGTTCATCA-3’ |
|  | Reverse: 5’-AATCCGTTCACACCGACCTT-3’ |
